# Temporally-resolved deep learning reveals autism symptom-specific neural signatures during naturalistic social experiences

**DOI:** 10.21203/rs.3.rs-9023571/v1

**Published:** 2026-04-22

**Authors:** Lei Li, Yuan Zhang, Anthony Strock, Saksham Pruthi, Srikanth Ryali, Vinod Menon

**Affiliations:** 1 Department of Psychiatry & Behavioral Sciences, Stanford University, Stanford, CA, United States.; 2 Department of Neurology & Neurological Sciences, Stanford University, Stanford, CA, United States.; 3 Wu Tsai Neurosciences Institute, Stanford University, Stanford, CA, United States.; 4 Stanford Institute for Human-Centered AI, Stanford University, Stanford, CA, United States.

## Abstract

Autism diagnosis lacks objective context-specific neurobiological markers, as traditional structural and resting-state neuroimaging fails to capture the dynamic social-cognitive processing differences that define the condition. We developed DualPathNet, an interpretable dual-stream deep learning network that simultaneously captures stable trait-like patterns and transient event-specific neural responses during naturalistic movie viewing. Across 555 children (274 ASD, 281 controls), our framework achieved > 70% accuracy using only 2-3 minutes of emotionally challenging stimuli, substantially outperforming 63% resting-state scans. Explainable AI with DualPathNet revealed that autism-related neural signatures were selectively expressed during high-demand social-emotional moments requiring empathy and emotion regulation, rather than uniformly expressed across all contexts. Critically, temporally specific neural responses during emotionally salient events predicted core autism symptoms including repetitive behaviors and social deficits. Our neuro-AI approach demonstrates that autism involves dynamic, context-dependent neural vulnerabilities rather than static disruptions, providing interpretable biomarkers for precision diagnosis and targeted intervention.

## Introduction

Autism spectrum disorder, a heterogeneous neurodevelopmental condition affecting more than 1 in 31 children worldwide ^1^, is characterized by difficulties in social communication and behavioral flexibility ^2,3^. Yet the dominant approaches for identifying its neural basis, relying primarily on structural MRI or resting-state fMRI that capture brain activity in the absence of social context, are inherently mismatched to the disorder’s defining features. Without engaging the social-cognitive processes central to autism, these paradigms have reinforced a static view that emphasizes trait-like disruptions in brain anatomy and network organization ^4-10^. This framework fundamentally fails to capture when and under what circumstances neural responses in individuals with autism diverge from neurotypical patterns during real-world social experiences, creating a critical gap between neurobiological findings and the dynamic, context-sensitive nature of autism as experienced by individuals and observed clinically. Here we address this mismatch by developing DualPathNet, a novel dual-stream deep learning architecture that simultaneously captures both persistent spatial and transient temporal aspects of brain function during naturalistic social experiences, revealing that autism-related neural signatures are not uniformly expressed but emerge selectively during emotionally challenging social moments.

Naturalistic paradigms using dynamic stimuli such as movies offer a novel approach that has the potential to align imaging methodology with the disorder's phenomenology ^11^. By presenting social narratives rich in emotional complexity, ambiguous intentions, and pragmatic communication demands, these paradigms have the potential to engage the very processes that distinguish autism: mentalizing, emotion recognition, social prediction, and flexible interpretation of verbal and non-verbal cues ^12,13^. Critically, the dynamic temporal structure of naturalistic stimuli such as movies enables researchers to identify not only where neural responses differ on average, but also when these differences emerge relative to specific social-emotional events. This temporal anchoring can transform autism neuroimaging from a search for static biomarkers to an investigation of dynamic vulnerabilities that manifest during high-demand social-emotional moments.

Despite the ecological advantages of naturalistic paradigms, their full potential remains unrealized. Most prior studies have relied on inter-subject synchronization analyses that, while useful for capturing shared neural engagement ^14,15^, inherently average across individuals and time, overlooking subject-specific and moment-to-moment variability critical for understanding heterogeneity in autism ^16,17^. Even recent machine learning and deep learning applications to autism neuroimaging have predominantly relied on temporally averaged features, such as static connectivity matrices or summary activation maps, thereby ignoring the fine-grained temporal dynamics that naturalistic data uniquely provide ^18^. As a result, rich temporal information is reduced to static summaries, obscuring when diagnostic differences emerge.

This methodological gap reflects a broader limitation: the lack of computational approaches that can jointly model spatial patterns and temporal dynamics while maintaining interpretability. Understanding autism’s context-dependent nature requires identifying not only which brain regions show atypical responses, but also when these responses diverge, which stimulus features they track, and how these spatiotemporal signatures relate to individual differences in clinical severity. Moreover, there is a pressing need for approaches that operate effectively under real-world conditions while yielding biologically meaningful and clinically actionable insights ^19,20^.

**Figure 1.**
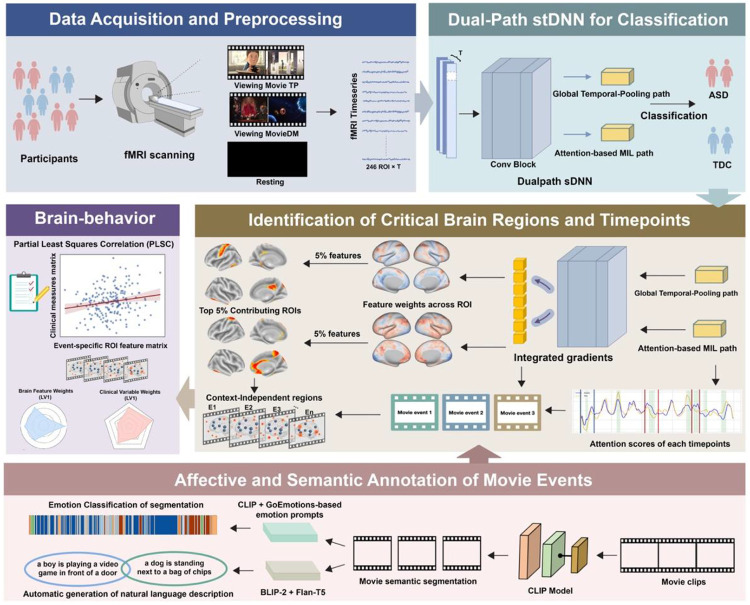
Dual-path deep neural network framework for ASD classification and interpretable brain feature discovery during movie viewing. Participants underwent movie-viewing and resting-state functional MRI (fMRI), from which preprocessed time series were extracted from 246 Brainnetome ROIs. A dual-path spatiotemporal deep neural network was trained to classify individuals with ASD versus typically developing controls (TDC). The model consisted of a GlobalMean path capturing stable, global spatial features and an attention-based multiple instance learning (MIL) path detecting temporally dynamic, event-level patterns. Events were identified using attention weights, and both cross-event and event-specific neural features contributing to classification were characterized. These neural features were further related to behavioral measures of symptom severity and cognitive performance. Movie events were automatically annotated using multimodal vision–language models (CLIP, BLIP-2, and Flan-T5) to enable semantic interpretation of temporally salient neural responses.

To address these challenges, we developed DualPathNet, a novel unifying dual-stream deep neural network architecture that simultaneously models the persistent and dynamic aspects of brain function (Figure 1). This framework is grounded in a fundamental hypothesis: that autism involves both stable, trait-like neural differences persisting across contexts and transient, event-specific disruptions emerging during moments of heightened social-emotional salience. These two types of neural signatures may contribute distinctly to the phenotype and associate with different symptom domains, requiring complementary analytical pathways to decode.

DualPathNet comprises two integrated streams that capture complementary aspects of the neural response. The Global Temporal-Pooling path summarizes spatial patterns over the entire viewing period by applying global average pooling across the temporal dimension, collapsing time while preserving ROI-level spatial features. This yields a trait-like representation of regional engagement, where “trait-like” refers to temporally stable, stimulus-wide neural patterns that persist across the viewing period rather than being tied to discrete events. Such representations resemble conventional time-averaged fMRI analyses. In contrast, the attention-based Multiple Instance Learning (MIL) path preserves full temporal resolution and employs frame-wise attention to identify transient neural responses linked to specific movie moments. Critically, this temporal pathway assigns different weights to individual time points, enabling the model to learn when neural activity is most diagnostically informative without prespecifying salient segments. Metadata covariates (age, sex, site) are incorporated into both paths to account for demographic and acquisition-related confounds. A learned fusion module then integrates global, time-averaged signals with local, event-level dynamics to produce the final classification.

We applied this framework to functional MRI data from the Child Mind Institute Healthy Brain Network (CMI-HBN), a large-scale, community-based developmental neuroimaging initiative featuring rich clinical phenotyping and naturalistic imaging paradigms ^21^. The dataset comprised more than 520 children (approximately 10-11 years old) per condition, making it the largest naturalistic fMRI sample currently available for autism research. Participants viewed two contrasting movie clips during scanning: excerpts from *Despicable Me* (movieDM), which alternate between humorous and emotionally negative scenes depicting both bonding and rejection, and a 3-minute animated film *The Present* (movieTP), which features emotionally negative and socially complex themes involving disability, social rejection, and empathy. These stimuli were selected to examine how variation in emotional valence and social complexity influences neural responses relevant to autism ^14,21,22^. The same cohort also completed resting-state fMRI scans, enabling a direct comparison between naturalistic and stimulus-free paradigms to evaluate whether ecological engagement enhances diagnostic classification.

DualPath was designed to address mechanistic questions about where, when, and under what conditions neural responses in autism diverge from those of typically developing individuals. We implemented multiple explainable AI (xAI) approaches to identify the most predictive brain regions and time points at both trait and event levels. For features captured by the Global Temporal-Pooling path, we identified the spatial patterns that consistently contributed to classification across the entire movie. For features captured by the attention-based MIL path, we identified specific temporal windows that carried the strongest predictive signal and then aligned these windows with the semantic and emotional content of the stimulus. To characterize those moments, we used a multimodal annotation pipeline combining vision-language models (CLIP, BLIP-2 ^23,24^) with text models (Flan-T5 ^25^) to generate segment-level semantic descriptions and assign fine-grained emotion labels based on the GoEmotions taxonomy ^26^. Together, these analyses allowed us to identify which types of social-emotional events – curiosity, empathy, disappointment, and social approach or avoidance – triggered the most divergent neural responses between the autism and control groups.

Our end-to-end AI framework provides mechanistic evidence that core autistic symptoms are rooted in the intensity of neural processing within multiple functional networks during specific moments of social salience. It uniquely integrates classification, interpretability, behavioral validation, and stimulus annotation to elucidate the spatiotemporal dynamics of atypical brain function in autism during naturalistic social experiences. By combining the ecological validity of movie-based fMRI with the analytical precision of temporally structured deep learning, our approach addresses a critical gap in developmental neuroimaging: moving beyond static, group-level contrasts to identify when, where, and under what emotional contexts individual autistic brains diverge from neurotypical processing.

## Results

### *Neural signatures in* movieTP *reveal temporally specific brain-behavior links*

#### DualPathNet achieves highest classification accuracy with emotionally challenging naturalistic stimulus

**Figure 2.**
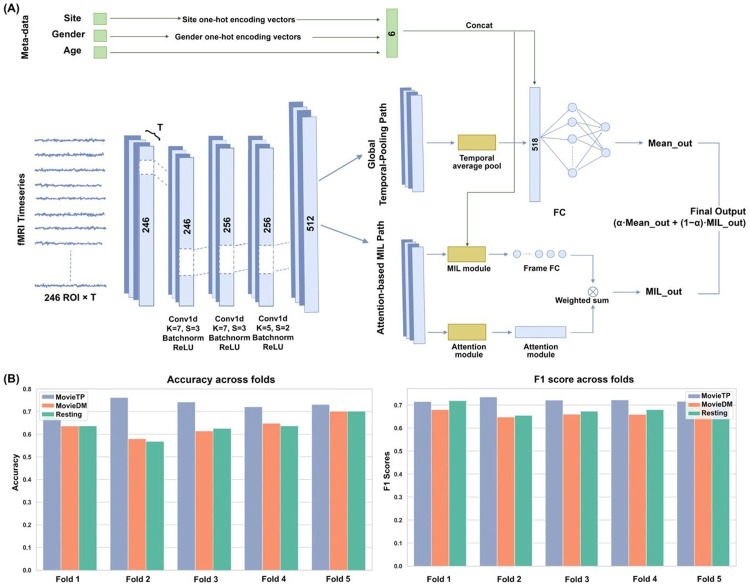
Model architecture and cross-validated classification performance. **(A) Network implementation.** The model takes region-wise fMRI time series and participant metadata (site, sex, age) as inputs. Temporal features are first processed by 1D convolutional layers and then routed into two branches: a Global Temporal-Pooling path and an attention-based MIL path. The branch outputs are fused to generate the final ASD vs. TDC classification. **(B) Classification Performance.** Bar plots show accuracy and macro-F1 scores across 5-fold cross-validation for three conditions: MovieTP, MovieDM, and Resting-state. The model achieves robust performance across tasks, demonstrating its generalizability.

We evaluated DualPathNet, a spatiotemporal deep neural network designed to model time-resolved neural representations, for ASD classification. The architecture comprises two complementary branches (Figure 2A): a Global Temporal-Pooling pathway capturing stable spatial patterns through temporal averaging, and an attention-based Multiple Instance Learning (MIL) pathway preserving full temporal resolution to identify transient, event-specific responses. Participant metadata (age, sex, site) are incorporated into both branches to account for these factors, and a learned weighted fusion mechanism integrates the two pathways to produce the final classification.

We applied DualPathNet to fMRI data acquired during viewing of *The Present* (movieTP), a cohesive 3-minute animated film featuring themes of disability, social rejection, and empathy. Using a nested cross-validation framework with stratified fivefold outer evaluation, the model achieved 73.1 ± 2.3% accuracy (macro-F1: 0.722 ± 0.008, precision: 0.713 ± 0.010, recall: 0.732 ± 0.012) (Figure 2B). Using the optimal hyperparameters identified via nested cross-validation, we repeated stratified fivefold cross-validation 100 times with different random splits. Additional controls, including site harmonization via ComBat, metadata orthogonalization, and comparisons with conventional machine learning models and prior deep learning architectures, consistently demonstrated superior DualPathNet performance (**Supplementary Results**; **Figure S1; Table S2**), indicating that the results are unlikely to be driven by methodological confounds.

#### Automated semantic and affective characterization of movie dynamics

**Figure 3.**
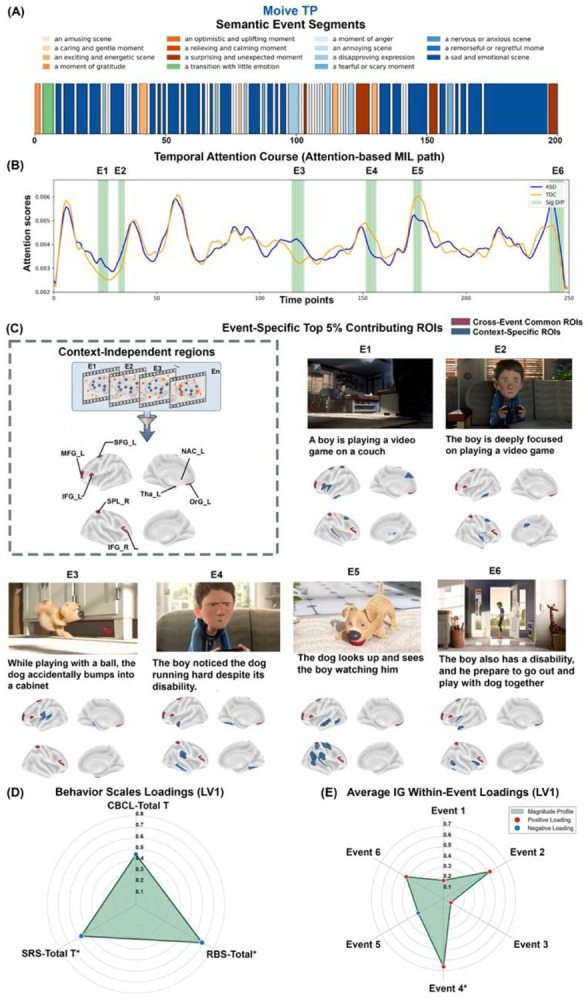
Spatiotemporal neural dynamics and brain-behavior mapping in *The Present* (MovieTP). **(A) Automatic semantic annotation via vision-language models.** A color barcode visualizes the affective landscape of the movie, automatically extracted by NLP models. **(B) Temporal attention dynamics.** Time course of model-derived attention scores for ASD (blue) and TDC (orange) groups. Green vertical bars mark six discriminative events (E1–E6) where significant group differences in neural saliency were identified (*p* < 0.05). **(C) Cross-event backbone and contextual-specific dynamics. Left (context-independent regions)**: schematic of backbone extraction, defined as regions appearing in ≥ 3 of the 6 discriminative events. The brain map displays cross-event common ROIs (consistent across events), highlighting a core social-valuation network involving the left inferior frontal gyrus (IFG_L), nucleus accumbens (NAC_L), thalamus (Tha_L), orbitofrontal gyrus (OrG_L), and superior frontal gyrus (SFG_L). **Right (event-specific top contributors)**: brain surfaces visualize event-specific ROIs (top 5% per event; E1–E6), aligned with representative screenshots, illustrating dynamic recruitment contingent on the specific narrative context. **(D-E) Multivariate brain-behavior associations (partial PLSC) in the ASD group.** Radar plots show loadings for the first latent variable (LV1). The neural profile is dominated by Event 4 (social loss), linking symptom severity (SRS/CBCL) to neural processing during empathy-eliciting moments. Asterisks (*) denote significant loadings (∣Bootstrap Ratio∣ > 1.96).

To objectively characterize the stimulus, we developed a multimodal annotation pipeline integrating PySceneDetect (temporal segmentation, https://github.com/Breakthrough/PySceneDetect), CLIP (zero-shot emotion classification), and BLIP-2 (natural language captioning). This analysis revealed that movieTP exhibits a cohesive, structurally integrated narrative organized into distinct, sustained temporal blocks, transitioning from scenes of curiosity and exploration to moments of realization and culminating in empathy-eliciting affective states (e.g., sadness, admiration, and confusion) (Figure 3A). This structured emotional progression supports both sustained, global neural engagement and temporally specific, event-level features relevant to diagnosis.

#### Global spatial features correlate with general cognition, but not core autism symptoms

Global Temporal-Pooling path analysis using group-averaged Integrated Gradients (IG) identified discriminative spatial features distributed across a broad social-cognitive and sensory-integration network. The top contributing regions across groups encompassed thalamus, inferior frontal gyrus, medial orbitofrontal cortex, and middle occipital gyrus (**Figure S2**). Detailed characterization revealed group-specific spatial patterns: ASD participants showed stronger reliance on thalamic and medial frontal regions implicated in internal evaluation and salience processing, whereas TDC participants engaged lateral temporal and parietal association areas involved in perceptual integration and social inference (**Table S3-S4**).

To test the behavioral relevance of these trait-like features, we derived a subject-specific Global Saliency Index by averaging IG magnitudes across the top 5% most discriminative regions. Partial correlation analyses controlling for age, sex, and site revealed a significant negative association with WISC Working Memory Index (WMI) Percentile (partial *r* = −0.145, uncorrected *p* = 0.049), along with marginal negative trends for Digit Span and Matrix Reasoning (partial *rs* ≈ −0.14, uncorrected *ps* < 0.06) (**Figure S3**).

Critically, no associations were observed with core autism symptoms assessed by the Social Responsiveness Scale (SRS), Repetitive Behavior Scale-Revised (RBS-R), or autism-relevant subscales of the Child Behavior Checklist (CBCL) (all *ps* > 0.05). This dissociation demonstrates that stable, trait-like spatial features are more closely related to general cognitive functioning than to symptom-specific neural dynamics, suggesting that distinct neural mechanisms may underlie different aspects of the autism phenotype.

#### Event-level dynamics reveal stable and phase-dependent contributors to ASD

To characterize event-level neural dynamics that differentiate ASD from controls, we leveraged the attention-based MIL path, which preserves full temporal resolution by assigning adaptive weights to individual time points. By analyzing the distribution of attention scores, we identified six discrete events (E1–E6) showing significant group differences (*ps* < 0.05), corresponding to distinct narrative beats: the boy's initial solitary play with a video game (E1–E2), his discovery of and interaction with the disabled dog (E3–E5), and the final revelation of the boy's own disability (E6) (Figure 3B). We validated the neural basis of the attention mechanism by computing time-resolved IG attributions and correlating them with attention scores, confirming robust temporal concordance (*rs* > 0.70, *ps* < 0.001; **Figure S4**).

To identify stable top contributors that persisted across changing narrative contexts in the ASD group, we constructed a Core Presence Matrix (**Table S5**) indicating whether each ROI appeared among the top 5% contributors for each event. Using this matrix as a spatiotemporal filter to quantify recurrence across events, we identified cross-event common regions that were consistently recruited despite dynamic shifts in the movie plot (e.g., from solitary play to interpersonal interaction) (Figure 3C**, Left**). The left inferior frontal gyrus exhibited the highest consistency, appearing in 5 out of 6 events, while the left nucleus accumbens, thalamus, and orbitofrontal gyrus appeared in 4 out of 6 events.

In addition to these stable contributors, we observed phase-dependent recruitment. The left superior and middle frontal gyrus were recruited exclusively during earlier narrative beats (E1 to E3), whereas the right superior parietal lobule appeared consistently during the later phase (E4 to E6). Each event also engaged context-specific regions (Figure 3C**, Right**). For example, the inferior parietal lobule and fusiform gyrus were uniquely recruited in E4, consistent with increased demands on social mentalizing and face processing. In contrast, earlier events such as E1 were characterized by engagement of anterior control hubs and subcortical sensory-gating regions. Together, these patterns indicate a spatiotemporal shift in event-specific recruitment from anterior executive-gating systems during solitary play toward posterior socio-visual hubs during interpersonal interaction.

To further characterize the functional involvement of the regions identified above, we mapped the ROIs onto the Dev-Atlas 24-network parcellation ^27^. At the network level, the cross-event common regions predominantly localized to the default mode and sensorimotor networks, suggesting a stable backbone supporting social valuation and sensory gating across events. In contrast, the phase-dependent regions reflected shifts within the dorsal attention and control networks, transitioning from anterior frontal nodes in earlier segments to more posterior parietal and temporal processing nodes later in the clip, as the narrative complexity increases.

#### Event-level dynamics pinpoint empathy recognition as mechanistic substrate of core symptoms

To further isolate event-level neural substrates specifically tracking core ASD symptomatology, we derived an event-specific Neural Saliency Index for each participant with ASD by averaging the absolute IG values within the top contributing regions identified for that event from the attention scores. This scalar metric quantifies the magnitude of discriminative neural recruitment, capturing how strongly the event-linked network pattern deviates from the neurotypical baseline during specific narrative moments. We then related individual saliency indices to the multidimensional clinical phenotype using partial least squares correlation (PLSC), controlling for age, sex, and site. Because the CMI-HBN cohort represents a large-scale transdiagnostic community sample and does not uniformly include gold-standard diagnostic instruments such as the ADOS, we used a targeted clinical-behavioral feature set comprising SRS Total, RBS-R Total, and selected CBCL subscales (Anxious/Depressed, Withdrawn/Depressed, Social Problems, Attention Problems) to capture core dimensions of social impairment, repetitive behaviors, and co-occurring psychopathology relevant to autism.

PLSC identified a significant multivariate mode (*r* = 0.340, *p* = 0.009) linking event-specific neural dynamics to a broad symptom cluster (**Figure S5A**). The behavioral loading profile was dominated by SRS Total (Weight = −0.569, BSR = −2.092, *p* = 0.037) and RBS-R Total (Weight = −0.687, BSR = −2.353, *p* = 0.019), capturing the core autism dimensions of social impairment and repetitive behaviors (Figure 3D).

The neural loading profile provided mechanistic insight into this brain-behavior relationship (Figure 3E). The association was driven primarily by E4 (Weight = 0.663, BSR = 2.102, *p* = 0.036), corresponding to the moment when the boy noticed the dog running hard despite its disability. E4 exhibited the strongest positive loading, indicating that greater neural saliency during this empathic recognition moment was associated with greater autism symptom severity. In striking contrast, other narrative moments, including initial solitary play (E1–E2, minimal loading) and the final revelation of the boy's own disability (E6, negative loading), contributed negligibly or inversely to the brain-behavior relationship.

This finding demonstrates that the severity of core autistic symptoms is related to the intensity of neural processing within the default mode, sensorimotor, and attention and control networks during a specific moment of recognizing and empathizing with another individual’s physical disability. The temporal specificity is remarkable: a brief (~10-second) narrative event accounts for substantially more symptom-related variance than the entire 3-minute session average, highlighting the power of event-level analyses to reveal context-dependent neural vulnerabilities.

### Neural signatures in *Despicable Me* confirm emotional valence effect

#### Rationale for analyzing emotionally negative segment

We next evaluated DualPathNet on a second naturalistic paradigm: excerpts from *Despicable Me* (movieDM), a 10-minute clip featuring the main character Gru and three orphan girls. The full clip achieved 63.6 ± 4.6% accuracy (macro-F1: 0.670 ± 0.023), substantially lower than movieTP despite longer scan duration (Figure 2B).

**Figure 4.**
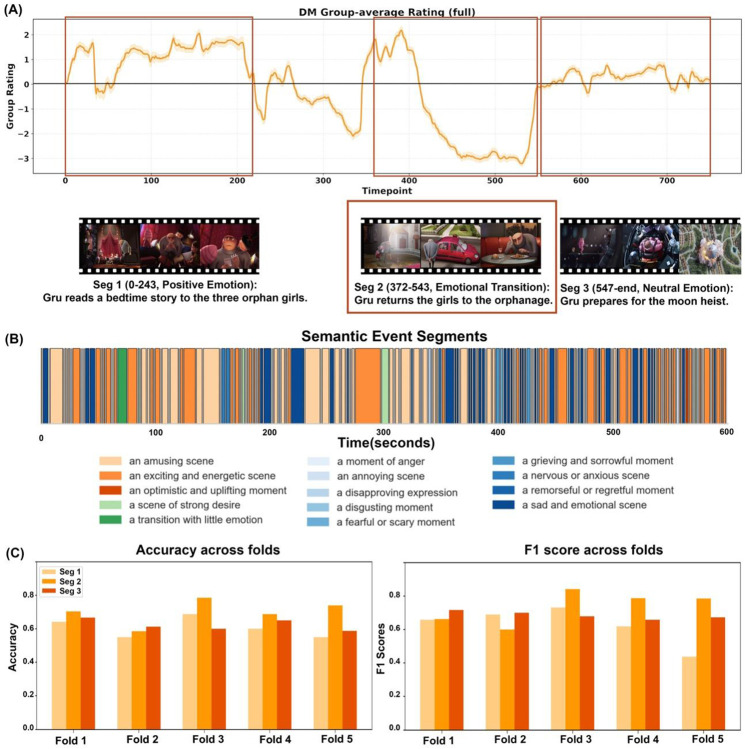
Performance validation and affective segmentation in *Despicable Me* (MovieDM). **(A) Human-annotated affective segmentation.** The group-average emotional rating trajectory splits the full movie into three distinct segments: Seg 1 (positive), Seg 2 (emotional transition), and Seg 3 (neutral). Seg 2 was selected for detailed analysis due to its high emotional variability. **(B) Automatic semantic event segmentation.** A fine-grained semantic barcode generated by vision-language models summarizes the narrative content of the MovieDM clip, enabling alignment of neural findings with objective, content-based semantic labels. **(C) Segment-wise performance.** Classification accuracy and macro-F1 scores across cross-validation folds are shown for the three segments, highlighting robust performance in the transitional segment (Seg 2).

To understand this performance difference, we characterized emotional structure of movieDM using our automated annotation pipeline together with independent human ratings of emotional valence ^28^. Twenty healthy adults (mean age = 25.0 ± 3.7 years) provided continuous pleasantness ratings on a −4 to +4 scale at 250-ms intervals across three viewings (**Figure S6**). Both AI-based emotion tagging and human ratings converged on the same conclusion: movieDM exhibits a highly heterogeneous, volatile emotional landscape with rapid shifts between conflicting affective states, from positive social bonding (Gru reading bedtime stories) to negative withdrawal and rejection (returning girls to orphanage) to neutral action sequences (moon heist preparation) (Figure 4A-B). The strong concordance between methods (*r* = 0.68, *p* < 0.001) validated this structural heterogeneity.

Given this mixed emotional content, we hypothesized that classification performance might be driven primarily by emotionally negative segments, consistent with our findings for movieTP. We therefore identified three coherent narrative segments based on converged emotional trajectory and story structure: Segment 1 (0–190s): Positive emotion—bedtime story reading and bonding; Segment 2 (298–435s): Emotional transition/negative—returning girls to orphanage, culminating in social separation; Segment 3 (438s–end): Neutral emotion—preparing for moon heist (Figure 4A-B).

Training DualPathNet separately on each segment revealed a clear emotional valence effect: Segment 2 achieved 70.1 ± 6.6% accuracy, approaching movieTP's performance and representing a dramatic ~10% improvement over the full clip. In contrast, Segments 1 (positive) and 3 (neutral) yielded only 60.6 ± 6.0% and 62.3 ± 3.4% accuracy, respectively (Figure 4C). These results demonstrate that, within a single movie stimulus, emotionally negative and socially challenging narrative moments carry substantially stronger diagnostic information than positive or neutral contexts.

We therefore focused subsequent interpretability analyses on Segment 2, where autism-related neural signatures were most pronounced. This choice was empirically justified: Segment 2 achieved the highest classification accuracy and showed the clearest separation between ASD and TDC neural representations.

#### Global spatial features capture group differences but not individual symptom variance

Using group-averaged IG, Global Temporal-Pooling path analysis of Segment 2 identified a distributed signature spanning the default mode and ventral attention networks. Top contributing regions in both groups included dysgranular insula, medial amygdala, perirhinal cortex, and dorsolateral prefrontal cortex (**Figure S7**), a network implicated in interoceptive awareness, emotional memory, and cognitive control during social processing. Detailed characterization revealed distinct group-specific weighting priorities within this shared circuitry (**Tables S6-S7**). ASD participants exhibited the strongest contributions from default mode network nodes (perirhinal cortex) and posterior dorsal attention network nodes (posterior parietal regions), regions involved in emotional memory retrieval and spatial attention to salient stimuli. In contrast, TDC participants showed relatively stronger engagement of lateral prefrontal control nodes and ventral premotor areas, suggesting greater reliance on top-down cognitive control and action simulation mechanisms when processing social rejection scenarios.

To test the behavioral relevance of these trait-level spatial features, we computed the Global Saliency Index (mean IG magnitude across top 5% discriminative regions) for each participant. Partial correlation analyses controlling for age, sex, and site revealed no significant associations with clinical symptoms (SRS, RBS-R, CBCL) or general cognitive performance (WISC) (all *ps* > 0.05).

This null result contrasts sharply with movieTP, where trait-level, time-averaged features were associated with working memory. The discrepancy likely reflects the emotional volatility of movieDM-Segment 2: even within this 2.5-minute segment, the narrative rapidly alternates between approach-related moments (e.g., Gru driving the girls to the orphanage) and avoidance-related moements (e.g., actual separation). As a result, the temporally pooled average may capture group-level diagnostic differences but dilute the stable, individual-specific variance needed to track behavioral traits. These findings suggest that under conditions of rapid emotional fluctuation, temporally resolved event-level analyses may be particularly important for revealing meaningful brain-behavior relationships.

#### Event-level dynamics reveal stable and event-specific contributors to ASD

**Figure 5.**
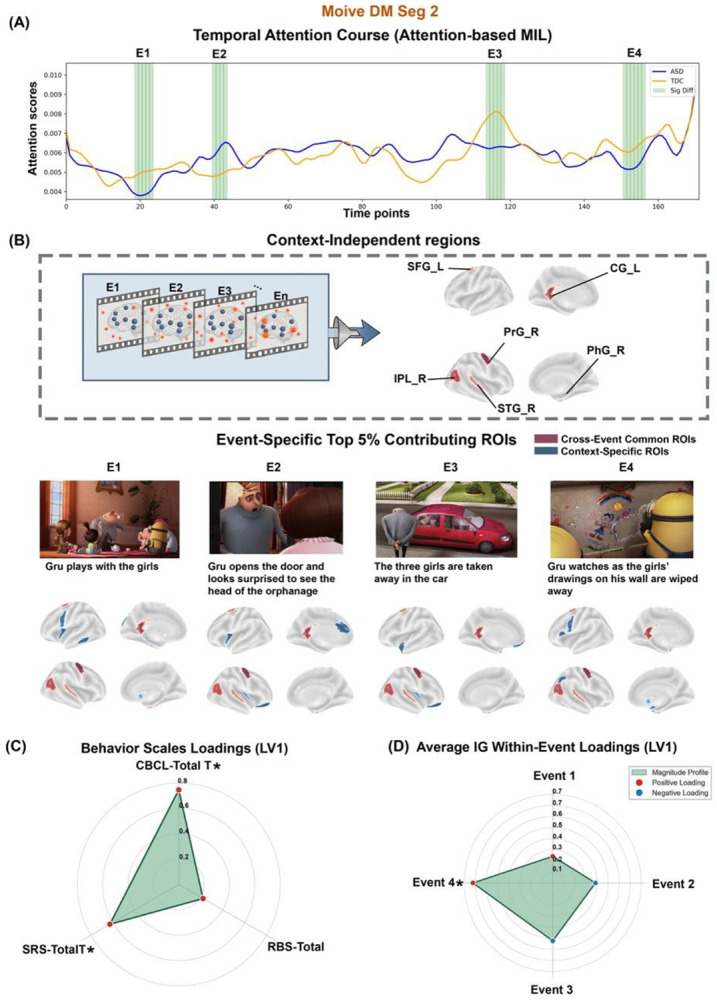
Spatiotemporal dynamics and brain-behavior mapping in *Despicable Me* (MovieDM, Segment 2). **(A) Temporal attention course.** Attention-score trajectories for the emotional transition segment, highlighting four discriminative events (E1–E4). **(B) Cross-event backbone and context-specific dynamics. Left (cross-event common ROIs)**: schematic of backbone extraction for this high-arousal segment, defined as regions appearing in 3-4 of the 4 discriminative events. This backbone consistently recruits dorsal attention, limbic, and sensory regions, including the superior frontal gyrus (SFG_L), precentral gyrus (PrG_R), parahippocampal gyrus (PhG_R), inferior parietal lobule (IPL_R), cingulate gyrus (CG_L), and superior temporal gyrus (STG_R). **Right (event-specific ROIs)**: event-specific maps show context-dependent recruitment patterns (e.g., amygdala or insula recruitment) aligned with representative screenshots (specific threats or social surprises). **(C-D) Multivariate brain-behavior associations.** Consistent with MovieTP, partial PLSC links symptom severity (SRS/CBCL) to event-specific neural responses, with the association driven primarily by **Event 4**, confirming the importance of emotionally salient moments in driving brain-behavior relationships. Asterisks (*) denote significant loadings.

The attention-based MIL path identified four discrete events (E1–E4) showing significant group differences in attention scores (*ps* < 0.05). These events aligned closely with Segment 2's emotional trajectory: the initial interaction at the orphanage entrance (E1), walking toward the building (E2), the moment of separation inside (E3), and the climactic moment when Gru watches the girls’ hand-drawn pictures being wiped away from his refrigerator representing profound social loss (E4) (Figure 5A).

Following the movieTP analytical framework, we validated concordance between attention scores and time-resolved IG attributions (*rs* = 0.74, *ps* < 0.001; **Figure S8**). We then identified the top 5% contributing regions for each event and computed event-specific Neural Saliency Indices for each participant with ASD. By examining the overlap of contributing regions across the four discriminative events within this emotional transition segment, we identified a robust cross-event common network (Figure 5B) from the Core Presence Matrix **(Table S8)**. A set of regions showed the highest consistency, appearing in all four events: the left superior frontal gyrus, right precentral gyrus, right parahippocampal gyrus, right inferior parietal lobule, and left cingulate gyrus. Additionally, the right superior temporal gyrus served as a supporting node, appearing in 3 out of 4 events. Functionally, under the Dev-Atlas parcellation, this cross-event backbone mapped predominantly to the default mode (DMN_2), dorsal attention (DAN_3), and sensorimotor (SMN_3) networks. Complementing these stable features, context-specific patterns demonstrated that neural recruitment was highly dynamic and event-dependent (Figure 5B). In contrast to the shared backbone, the top contributing regions varied substantially across the four discriminative events, yielding distinct spatial configurations for each moment rather than a uniform network topology. Notably, while the stable backbone was anchored in dorsal-posterior regions, event-specific recruitment, particularly during high-arousal moments such as Event 4, shifted towards affective nodes within the default mode (e.g., amygdala) and control networks, reflecting a specific engagement of emotion-regulation processes during social stress.

#### Event-level dynamics reveal social loss as key driver of symptom associations

We employed PLSC to link event-specific neural dynamics with clinical phenotype, using the same behavioral feature set as in movieTP (SRS, RBS-R, CBCL subscales) and controlling for age, sex, and site. This analysis revealed a significant multivariate mode (*r* = 0.48, *p* = 0.035) (**Figure S5B**). The behavioral loading profile was dominated by CBCL Total (Weight = 0.747, BSR = 2.319, *p* = 0.020) and SRS Total (Weight = 0.628, BSR = 2.202, *p* = 0.028), indicating a symptom axis spanning generalized psychopathology and social impairment—a slightly broader symptom profile than movieTP’s focus on social and repetitive behaviors (Figure 5C).

Critically, the neural loading profile revealed striking temporal specificity mirroring the movieTP findings (Figure 5D): associations were distinctively driven by Event 4 (Weight = 0.708, BSR = 1.988, *p* = 0.049), the scene depicting social loss and the erasure of the girls’ drawings. This moment showed the highest positive loading, indicating that greater neural engagement during processing of profound social rejection predicts more severe autism symptoms. Event 1 (initial neutral interaction at the orphanage entrance) contributed minimally, and intermediate events (E2–E3) showed intermediate contributions.

These results demonstrate that brain-behavior relationships in ASD are linked to the strength of neural processing within the default mode, sensorimotor, attention and control networks during specific moments of social-emotional loss, not distributed across positive or neutral social interactions. The convergence with movieTP, despite completely different narrative content (disability/empathy vs. rejection/loss), stimulus structure (cohesive vs. fragmented), and duration (3 min vs. 2.5 min segment), provides robust evidence for this temporal specificity.

### Resting-state shows weaker performance and no clinical associations

#### Stimulus-free paradigm yields lower classification accuracy

To directly evaluate whether naturalistic engagement enhances diagnostic classification relative to stimulus-free conditions, we applied DualPathNet to resting-state fMRI data from the same cohort. Participants were instructed to rest quietly with eyes open while fixating on a central crosshair for approximately 10 minutes. Using matched 3-minute segments, and identical preprocessing, feature extraction, and cross-validation procedures, the model achieved 63.32 ± 4.73% accuracy (macro-F1: 0.688 ± 0.027) (Figure 2B).

This performance falls ~10 percentage points below both movieTP (73.14%) and movieDM-Segment 2 (70.06%), the two emotionally challenging naturalistic conditions. The consistency of this performance hierarchy across multiple cross-validation runs and control analyses indicates that emotionally negative naturalistic stimuli provide a robust and replicable advantage over resting-state fMRI for ASD classification.

#### Absence of behavioral correlations suggests limited clinical utility

We computed the Global Saliency Index for resting-state data and performed comprehensive partial correlation analyses controlling for age, sex, and site across the full behavioral battery: SRS, RBS-R, CBCL subscales, and WISC indices. No significant associations were observed between resting-state neural features and any clinical or cognitive measures (all *ps* > 0.05, uncorrected).

## Discussion

Understanding autism through neurobiological signatures has been constrained by a fundamental methodological mismatch: we have studied a disorder of dynamic social cognition using paradigms that eliminate social context ^29^. Our study establishes a novel framework by integrating naturalistic movie-viewing with temporally structured deep learning, revealing that autism-related neural signatures are not static or uniform but instead dynamic and context-dependent, emerging selectively during high-demand social-emotional moments.

We make three fundamental contributions. First, we demonstrate that emotionally challenging content reveals autism-related neural differences far more effectively than positive or resting-state paradigms, achieving higher classification performance with just three minutes of naturalistic viewing ^17^. This challenges assumptions of uniform neural disruption and reveals that vulnerabilities are preferentially detected during moments requiring emotion regulation and social inference ^30^. Second, we show that autism comprises dissociable global and event-specific neural mechanisms with distinct behavioral correlates. Stable spatial patterns were associated with general cognitive functioning, but not autism symptoms. In contrast, transient temporal dynamics, occurring during specific moments of empathy recognition or social rejection, predicted core autism symptoms including repetitive behaviors and social difficulties. This provides neurobiological validation for multi-dimensional models of the disorder. Third, we provide an interpretable framework that identifies which brain regions, temporal moments, and social-emotional contexts drive individual classification, enabling personalized neural signatures rather than group-level statistics.

These findings challenge the field’s reliance on static connectivity frameworks and suggest that brief, ecologically valid paradigms may provide richer mechanistic insights than traditional approaches ^31^. This work offers methodological foundations for a path toward personalized, biologically grounded AI models that capture when and how neural responses in individuals with autism diverge during real-world social experiences.

### The emotional valence effect: context-dependent neural vulnerabilities

A striking finding from our study is that emotionally negative and socially complex content revealed autism-related neural signatures with substantially greater fidelity than positive or neutral content. Using just three minutes of viewing time, the film *The Present*, depicting disability and empathy, achieved 73% accuracy, while the emotionally negative segment of *Despicable Me*, depicting social rejection and loss, achieved 70%, both substantially outperforming resting-state scans (63%). The convergence across two independently analyzed stimuli with different structures strengthens this conclusion. *The Present* features a cohesive narrative arc building toward empathic realization, while *Despicable Me* Segment 2 depicts rapid emotional transitions culminating in social loss. Despite these structural differences, both emotionally negative contexts achieved similar superior performance (~70-73%), while positive bonding scenes in *Despicable Me* Segment 1 achieved only ~61%, barely above resting-state. These results provide novel evidence that autism involves dynamic, context-dependent neural vulnerabilities that are preferentially revealed by emotionally challenging and socially ambiguous naturalistic content rather than positive social interactions or stimulus-free conditions.

This emotional valence effect has profound theoretical implications. If autism involved uniform disruptions in brain connectivity or social processing, we would expect similar classification performance across all movie conditions and resting-state scans. Instead, the dramatic performance advantage of negative emotional content indicates that autistic brains diverge most significantly from neurotypical patterns under specific contextual demands. Scenes depicting emotional distress, interpersonal rejection, or ambiguous social intentions engage systems involved in emotion regulation, mental state attribution, and salience prioritization ^32,33^. Our findings suggest disrupted coordination or atypical prioritization of these systems, resulting in temporally specific neural divergence.

Our findings suggest that naturalistic paradigms can differentiate autism more effectively than traditional paradigms. This has potential implications for early identification: rather than relying on lengthy resting-state scans that may miss critical developmental signatures, brief exposures to emotionally salient social narratives could provide more sensitive and specific biomarkers.

### Temporal event dynamics distinguishing autism

The attention-based MIL pathway revealed temporally focal moments when individuals with autism diverged most dramatically from neurotypical responses ^34^. Leveraging these moments, we show that the neural phenotype of autism reflects a hierarchical architecture: a stable neurobiological backbone overlaid by transient, context-specific dynamics, each associated with complementary xAI-derived brain features ^19,20^.

In movieTP, the most discriminative moment occurred when the protagonist empathizes with a disabled dog. Underlying this narrative shift, we identified a robust, cross-event backbone anchored in the left inferior frontal gyrus and a subcortical loop involving the nucleus accumbens and thalamus. The persistence of these regions suggests that individuals with autism maintain a tonic mode of internal valuation (DMN) coupled with rigid sensory gating (SMN) throughout the social narrative. This pattern contrasts with the neurotypical tendency to flexibly modulate the DMN engagement in response to contextual demands ^35^. Notably, during the high-empathy event, this stable core did not account for the observed divergence. Instead, the autism group exhibited a spatial shift within the dorsal attention and control networks, transitioning from anterior frontal nodes (early phase) to posterior parietal nodes (late phase). This temporal progression suggests that individuals with autism may rely less on the typical DMN simulation route ^36^. Together, these findings suggest a dual-process mechanism in autism: a rigid, tonic DMN-SMN backbone that operates continuously, coupled with phasic anterior-to-posterior attentional reallocations that are only triggered when social-emotional demands exceed a critical threshold ^37,38^.

In movieDM, the diagnostic focus converged on scenes depicting social loss and rejection. Here, the stable backbone was functionally distinct and comprised dorsal attention (e.g., superior frontal gyrus) and default mode (e.g., parahippocampal gyrus) nodes. This pattern indicates a baseline effort to maintain cognitive control and scene maintenance during high-arousal transitions ^39^. Yet, the autism group showed a specific breakdown of this dorsal pattern during the moment of rejection, characterized by heightened neural saliency in affective nodes within DMN (specifically the amygdala) and the VAN (subgenual anterior cingulate) ^40^, pointing to aberrancies in emotion regulation during social rejection. This observation provides a mechanistic basis for the emotion regulation difficulties frequently reported in autism ^41^ and supports the theory that social impairments may stem from an imbalance between heightened bottom-up affective reactivity and insufficient prefrontal modulation ^42^.

Critically, neural responses during these specific events were correlated with core autism symptoms including repetitive behavior and social difficulties while global features did not show such associations ^43,44^. Temporally averaged spatial signatures were not correlated with core autism symptom severity on the SRS or RBS-R in either viewing condition. Even in movieTP, where global features were linked to working memory performance, they remained dissociated from diagnostic symptom scores. This double dissociation suggests that the Global Temporal-Pooling pathway indexes a categorical marker of the diagnosis but lacks sensitivity to individual symptom variation ^45,46^, limiting their utility for tracking treatment response or predicting outcomes.

This dissociation provides a crucial advance over prior naturalistic fMRI studies, including those using the same CMI-HBN autism dataset, which employed inter-subject synchronization to map shared processing ^47^. While these studies established that autism is characterized by idiosyncratic brain activation patterns that deviate from the synchronized responses observed in neurotypical groups ^48^, they aggregate data across the entire movie duration.

Our temporally-resolved findings demonstrate that this neural idiosyncrasy is not static or uniform. Instead, we show that the breakdown in neural processing in naturalistic social contexts, and its direct link to symptom severity, is phasic, driven by specific 'stress tests' within the narrative encompassing moments of rejection or empathy ^49^. This supports the 'contextual idiosyncrasy' hypothesis ^50^: brains of individuals with autism diverge from the neurotypical consensus primarily when the social-emotional demands exceed a critical threshold, rather than functioning atypically across all contexts.

### Neural gain control failures: a dynamic dysregulation framework

Our findings are noteworthy from a brain dynamics perspective suggesting that autism may involve disruptions in neural "gain control", the ability to dynamically modulate response magnitude based on contextual demands ^51^. During emotionally neutral or positive scenes, neural responses in individuals with autism may function similarly to those of typically developing individuals. However, during emotionally challenging moments requiring rapid integration of multiple social cues, flexible perspective-taking, and emotion regulation, neural coordination may break down. The attention mechanism in our MIL pathway effectively identifies these moments of breakdown by learning which time points maximize diagnostic separation between groups.

Importantly, the ability to identify when neural differences occur, not just where, opens new possibilities for mechanistic investigation. Future studies could systematically manipulate the temporal structure of naturalistic stimuli by varying the pacing of emotional events, the predictability of social outcomes, and the complexity of concurrent cues, to test hypotheses about what specific processing demands trigger neural divergence.

### DualPathNet with LLM-based annotation: advancing interpretable AI frameworks for time-resolved, individual-level neural and contextual signatures

More broadly, our study provides a novel interpretable AI framework for identifying which brain regions, temporal moments, and social-emotional contexts drive individual classification, enabling person-specific neural signatures. For each individual, our explainable AI framework provides three complementary levels of interpretability. At the spatial level, Integrated Gradients identified which specific brain regions contribute most to distinguishing autism from neurotypicals. At the temporal level, attention weights from the MIL pathway revealed which moments during the movie drive diagnostic certainty. At the contextual level, our automated annotation pipeline aligns these neural responses with semantic and emotional stimulus features.

This multi-scale approach enables us to determine not just that an individual’s brain differs from the neurotypical pattern, but how it differs, when these differences are most pronounced, and what kinds of social-emotional situations elicit atypical responses.

The integration of automated stimulus annotation with interpretable deep learning creates a powerful closed loop for mechanistic discovery. Using computer vision models (CLIP) and large language models (BLIP-2, Flan-T5), we extracted frame-level natural language descriptions and fine-grained emotional labels from the GoEmotions taxonomy for each video segment. This computational approach objectively quantifies the semantic landscape of naturalistic stimuli, determining precisely how narrative and emotional content unfolds over time at the fMRI timescale. Critically, this automated framework was validated against continuous human emotion ratings for *Despicable Me*, with AI-generated labels showing high concordance with human-perceived affective structure, establishing biological validity while maintaining scalability and reproducibility.

When the attention-based MIL pathway identified diagnostically informative time points, the annotation system revealed what made those moments distinctive. For example, in *The Present*, Event 4, where attention weights peaked and IG attribution was maximal, corresponded to the moment “the boy noticed the dog running hard despite its disability,” tagged with emotions of "admiration," "realization," and "sadness." Spatial analysis of this specific event revealed that the autism group recruited a unique neural circuit. Similarly, in *Despicable Me* Segment 2, Event 4 captured "watching the girls' drawings being wiped away", a moment of profound social loss with the autism group showing distinct neural signatures.

This event-specific brain mapping demonstrates that diagnostic neural signatures are not uniformly distributed but emerge during specific narrative moments requiring particular cognitive-emotional operations. By linking attention-identified events to both their semantic content (via LLM-generated descriptions) and their neural substrates (via IG attribution), our framework enables hypothesis generation about mechanism. The finding that diagnostic moments cluster during scenes involving "disappointment," "remorse," and "anxiety", rather than simpler negative emotions like anger or sadness, suggests autism involves specific difficulties with self-conscious emotions requiring social evaluation and perspective-taking ^49,50^.

Importantly, these individualized neural profiles correlate with symptom severity: reduced activation during these specific events predicted greater repetitive behaviors and social difficulties, while global spatial patterns did not. This demonstrates that person-specific, event-level neural signatures carry clinically meaningful information about individual variation in autism symptomatology, opening possibilities for personalized interventions targeting the specific contexts where each child shows neural vulnerability.

## Conclusion

This work establishes a novel AI-driven paradigm for understanding autism by demonstrating that its neural signature is not static and uniform but dynamic and context-dependent, emerging selectively during emotionally challenging social moments that require flexible regulation and integration. By integrating naturalistic movie-viewing paradigms with temporally structured, interpretable deep learning, we show that brief, ecologically valid assessments can outperform traditional resting-state approaches while providing richer mechanistic insights and individual-level precision.

Our findings advance the field beyond static connectivity frameworks toward dynamic models that capture when and under what conditions autistic brains diverge from neurotypical patterns. The identification of specific social-emotional contexts that reveal vulnerabilities, and the demonstration that different neural mechanisms underlie different symptom domains, opens new possibilities for personalized intervention targeting context-specific difficulties rather than treating autism as a monolithic condition. As neuroimaging moves toward clinical translation and precision medicine, frameworks that combine ecological validity, temporal precision, interpretability, and individual specificity will be essential. This study provides methodological foundations, proof-of-concept evidence, and a conceptual framework for such integrative approaches not only for autism but for developmental neuropsychiatry more broadly.

## Methods

### Study Cohorts and Participants

We utilized functional MRI data from the Child Mind Institute Healthy Brain Network (CMI-HBN), a large-scale, community-based dataset comprising multimodal neuroimaging and structured clinical assessments. ASD diagnoses were determined through a standardized, multi-stage process that combined semi-structured interviews and expert clinical review. Specifically, all participants underwent the KSADS-COMP (Kiddie Schedule for Affective Disorders and Schizophrenia–Computerized Version), a DSM-5-aligned diagnostic interview administered by licensed clinicians. Algorithm-derived diagnoses from the KSADS-COMP were reviewed alongside other clinical materials, and final DSM-5 consensus diagnoses were assigned by licensed psychologists or psychiatrists (see Supplementary Methods for full diagnostic protocol and inclusion criteria) ^21^.

For the present study, we included participants with confirmed or presumptive ASD diagnoses and those without any psychiatric diagnoses as TDC. Inclusion further required completion of both resting-state and two naturalistic movie-viewing fMRI runs on a 3T scanner. The initial preprocessed sample included:

- Despicable Me (DM): 258 ASD and 261 TDC participants,
- The Present (TP): 274 ASD and 281 TDC participants,
- Resting-State: 297 ASD and 275 TDC participants,

During scanning, participants passively viewed two short movies as part of the functional imaging protocol. The Despicable Me clip (00:10:00 duration) was extracted from the full-length feature film (Despicable Me 1) and presented from timestamp 01:02:09 to 01:12:09 (https://despicableme.fandom.com/wiki/Despicable_Me_(film)), while The Present (00:03:21 duration) was presented from 00:00:00 to 00:03:21 (https://www.youtube.com/watch?v=3XA0bB79oGc). Precise stimulus timing and synchronization were implemented via PsychoPy scripts provided by the data consortium.

Details of the MRI acquisition and task presentation procedures are available at the CMI-HBN MRI Protocol website (https://fcon_1000.projects.nitrc.org/indi/cmi_healthy_brain_network). The final analyzed sample sizes and demographic characteristics are reported in Table 1. Characteristics of the full initial cohort prior to exclusion are provided in **Table S1**.

### fMRI Preprocessing and Feature Extraction

fMRI data were preprocessed using SPM12 and custom scripts (see *Supporting Information: fMRI Preprocessing*). Standard steps included realignment, co-registration with T1 images, normalization to the MNI152 template, and smoothing. Time series were band-pass filtered (0.008–0.09 Hz), and nuisance variables including head motion, white matter, and CSF signals were regressed out. ROI-level signals were extracted using the Brainnetome Atlas (246 ROIs; ^52^). To account for the hemodynamic response delay, a 4-second temporal shift was applied to align brain responses with movie content. Final training and evaluation cohorts were determined after standard preprocessing steps and exclusion criteria (e.g., head motion FD > 0.5 mm, >10% volumes repaired, data shape mismatch, or NaNs). The resulting sample sizes and demographic characteristics are reported in **Supplementary Table S1**.

### DualPathNet Model Architecture

We implemented a DualPath spatiotemporal deep neural network (DualPathNet) model to classify ASD and TD participants based on fMRI time series. The architecture consists of two parallel branches: (1) The Global Temporal-Pooling path captures global spatiotemporal patterns using average pooling; and (2) The attention-based MIL path detects transient, event-level features using frame-wise attention. Both branches share a 1D convolutional backbone (kernel sizes = 7, 7, 5) followed by BatchNorm, ReLU, and Dropout (0.5). The input shape is B × C × T (batch size × 246 ROIs × timepoints). Metadata—sex (2 dummy vars), site (3), and age (1)—are concatenated to the feature representations in both branches (see *Supporting Information: DualPathNet Architecture*). The outputs from both branches are fused using a weighted sum, with weight *α* controlling the contribution of each path. The final class label is determined by taking the argmax of the fused logits. Model training minimizes the cross-entropy loss.

### Model Training and Cross-Validation

Model training was implemented in PyTorch Lightning (https://lightning.ai/), with Weights & Biases (https://wandb.ai/site) used for experiment tracking and monitoring. To obtain an unbiased estimate of model generalization performance, we employed a nested cross-validation framework. Specifically, stratified fivefold outer cross-validation was used to evaluate model performance while preserving balanced group distributions. To ensure subject-level independence, fold assignments were generated using stratified group-based splitting, preventing samples from the same subject appearing in both training and test sets. Within each outer training fold, hyperparameter optimization was conducted using an inner cross-validation loop. Hyperparameters—including learning rate, batch size, dropout rate, and fusion weight α—were sampled from predefined ranges using random search. For each candidate configuration, model selection was based on mean validation accuracy across inner folds.

After selecting the optimal hyperparameters, the model was retrained on the full outer training fold (with a small internal holdout set used for early stopping) and evaluated exclusively on the corresponding outer test fold. Early stopping was based on validation accuracy with a fixed patience parameter. Final performance was reported as the mean and standard deviation of accuracy, precision, recall, and macro-F1 score across outer folds

### Brain Feature Attribution and Interpretation

To decrypt the deep learning model’s decision-making and identify key spatiotemporal drivers of classification, we employed a path-specific interpretability framework leveraging IG ^53,54^ and attention-based saliency mapping.

For the Global Temporal-Pooling path, which captures time-invariant neural recruitment, we applied IG to the temporally pooled representation to identify stable spatial signatures, computing robust group-level attribution maps by selecting the top 5% of ROIs with the highest absolute IG values to define a Trait-Level saliency map of intrinsic networks.

Conversely, for the time-resolved MIL path, we adopted a validation-then-characterization strategy to resolve temporal heterogeneity. Discriminative events were first rigorously defined as continuous time windows where group-level attention scores differed significantly (*p* < 0.05). Crucially, to validate that these attention weights reflected genuine neural recruitment, we calculated the Pearson correlation between the time-resolved global mean IG attributions and the model’s attention scores; the observed high temporal concordance confirmed that the attention mechanism served as a reliable proxy for neural saliency. Leveraging this validation, we characterized the specific neural topography for each defined event by computing the median absolute IG value within the event window and selecting the top 5% ROIs.

To identify the stable neurobiological backbone underlying the dynamic narrative, we constructed a Core Presence Matrix based on the recurrence of these top-contributing regions. We defined core common regions as ROIs that appeared in the top 5% contributing set in the majority of events (e.g., ≥ 3 out of 6 events for movieTP, or ≥ 3 out of 4 events for movieDM Segment 2). Conversely, context-specific regions were defined as those appearing uniquely in specific event windows. To facilitate developmentally accurate functional interpretation, all identified Brainnetome ROIs were mapped to the Dev-Atlas 24-network parcellation ^27^ using a maximal spatial overlap strategy. Specifically, voxel-wise spatial overlap was computed between each Brainnetome ROI and all Dev-Atlas parcels, and each ROI was assigned to the network with the highest proportion of overlapping voxels. Detailed implementation procedures are provided in the **Supplementary Methods**. This allowed us to categorize regions into specific functional subnetworks (e.g., DMN_4, SMN_3) within six canonical systems (Default Mode, Sensorimotor, Visual, Control, Dorsal Attention, and Ventral Attention).

Finally, to quantify the intensity of discriminative neural recruitment for downstream analysis, we derived a subject-specific Neural Saliency Index for both pathways by averaging the absolute IG values within their respective top-contributing regions.

### Multivariate Brain-Behavior Mapping

To bridge the model-derived neural signatures with the multidimensional clinical phenotype of the transdiagnostic CMI-HBN cohort, we implemented a robust statistical framework. Given the extensive set of behavioral measures available in the CMI-HBN dataset, we selected a subset of 29 items from four representative instruments based on two criteria: (1) theoretical relevance to autism core domains (e.g., cognition, social communication, repetitive behaviors), and (2) availability across majority of participants. These included: CBCL (Child Behavior Checklist): cognitive and behavioral regulation; WISC (Wechsler Intelligence Scale): visual processing and spatial intelligence ^55^; SRS (Social Responsiveness Scale): social communication and reciprocity ^56^; and RBS (Repetitive Behavior Scale): repetitive and restricted behavior traits ^57^.

All behavioral scores were either standardized T-scores or normalized scale scores to ensure comparability across participants. To account for potential confounders, we rigorously controlled for age, sex, and acquisition site across all analyses. We employed a dual-branch statistical strategy tailored to the distinct nature of the neural features:

#### Global Path:

For the time-invariant Global Temporal-Pooling path, which captures stable global signatures, we examined the linear association between the Global Saliency Index (mean IG of top 5% regions) and behavioral scores. Partial Pearson correlation analyses were performed to quantify the strength of these associations while strictly regressing out the effects of nuisance covariates.

#### MIL Path:

For the time-resolved MIL path, we employed Multivariate Partial Least Squares Correlation (PLSC) to investigate the complex multivariate brain-behavior interface. Variable selection was conducted rigorously to ensure model stability and interpretability. First, while the sample size was robust (N ≈ 200), including the full spectrum of individual subscales would result in a behavioral feature space that is disproportionately high-dimensional relative to the compact set of event-level neural indicators (only 4–6 events). This dimensional imbalance between the brain and behavioral matrices could compromise the model's ability to extract symmetric latent structures and increase the risk of overfitting. Second, preliminary univariate analyses revealed that general cognitive measures (WISC indices) were not significantly associated with the dynamic event-specific saliency indices (*p* > 0.05). Consequently, we restricted the behavioral input matrix to a parsimonious set of total scores from the three instruments most robustly indexing core autism symptomatology: SRS-Total (social), RBS-Total (repetitive), and CBCL-Total (general psychopathology). Prior to analysis, all Neural Saliency Indices underwent Rank-Based Inverse Normal Transformation (Rank-INT) to mitigate outliers and ensure normality. PLSC utilizing Singular Value Decomposition (SVD) was then applied to these targeted matrices to identify orthogonal Latent Variables (LVs) maximizing the covariance between dynamic neural processing and symptom severity. The statistical significance of the identified LVs was assessed via permutation testing (5,000 iterations). Crucially, to ensure the reliability of feature contributions, we performed bootstrap resampling (5,000 iterations) to compute Bootstrap Ratios (BSR) for each brain and behavioral weight. Features with a BSR corresponding to ∣z∣ > 1.96 (*p* < 0.05) were considered robust and stable contributors to the brain-behavior association.

### Manually annotated emotional content and narrative event segmentation

Because MovieDM (~10 min) is substantially longer and narratively richer than MovieTP (~3 min), to characterize the emotional structure of MovieDM and to obtain analysis-ready time windows, we used human valence annotations from prior work ^28^ and performed an additional narrative-driven re-segmentation to isolate complete events rather than arbitrary windows. We applied a two-stage procedure: Valence time series (1–9 scale; 1 = most negative, 9 = most positive) were standardized within rater and averaged across runs and raters to obtain a group emotional-valence vector per TR (**Figure S6**). We then aligned this vector to cinematic scene boundaries (hard cuts and narrative beats) and defined segments that captured self-contained narrative units with coherent emotional meaning. Boundaries were placed at natural shot/scene transitions to preserve event integrity and to avoid splitting actions across segments.

This procedure yielded three emotionally and narratively distinct segments (TR indices in parentheses): **Segment 1** (0–243; Positive Emotion), Gru reads a bedtime story to the three orphan girls; **Segment 2** (372–543; Emotional Transition/Negative turn), Gru returns the girls to the orphanage; **Segment 3** (547–end; Neutral/Action-oriented), Gru prepares for the moon heist.

The segments above were used to time-lock neural responses, constrain attention-weighted frame selection, and ensure that dynamic effects reflect event-level processing rather than arbitrary temporal windows.

### Video Annotation and Alignment with Neural Data

To enhance the interpretability of neural responses during naturalistic movie viewing, we developed a multi-level annotation framework aimed at linking fMRI-detected neural discriminative moments to meaningful semantic and emotional content in the stimuli. This framework allowed us to understand what types of events or emotions in the movie contributed to ASD vs. TDC classification. The annotation pipeline included the following components:

#### Scene Segmentation:

We used PySceneDetect (https://github.com/Breakthrough/PySceneDetect) to identify scene boundaries based on visual transitions and shot changes. This provided a basic structure for temporal units of the stimulus.

#### Semantic Clustering:

Frame-level embeddings were generated using the CLIP model ^23,58^, and unsupervised clustering was applied to categorize semantically similar events into distinct event types (e.g., social interactions, object manipulation).

#### Emotion Tagging:

A zero-shot classification approach was implemented using CLIP and 30 emotion labels from the GoEmotions taxonomy ^26^This allowed us to assign fine-grained affective labels (e.g., excitement, sadness, curiosity) to each movie segment without requiring manual labeling ^59^.

#### Captioning:

To facilitate qualitative understanding, we used the BLIP-2 and Flan-T5-XL models ^25^ to generate natural language descriptions of each segment, providing intuitive context for behavioral interpretation.

#### Temporal Alignment:

All segment-level annotations were temporally aligned to fMRI signals with a 4-second hemodynamic delay to account for BOLD response lag. These annotations were cross-referenced with IG-derived and attention-based discriminative time points, allowing us to link brain-level classification signals to specific semantic/emotional events in the movie.

This annotation strategy provided a structured bridge between computational model outputs (i.e., neural attribution maps) and the ecological richness of the stimulus, enabling event-level decoding of social and affective features that distinguish ASD from TDC participants. See **Supporting Information: Video Annotation Alignment** for further methodological details and examples. A complete list of annotated video segments—including emotion labels and natural language descriptions—for both movie clips (TP and DM) is provided in the **Supplementary Materials** (see CSV files under “Video Annotation and Alignment”).

## Supplementary Material

This is a list of supplementary files associated with this preprint. Click to download.

- SupportingInformation.docx

## Figures and Tables

**Table 1.** Final demographic and head-motion characteristics after quality control and exclusions. Summary statistics for sample size (N), sex distribution (M/F), mean age, and mean framewise displacement (FD) for participants with ASD and TDC across three fMRI conditions: MovieDM, MovieTP, and resting state.

Movie DM				
Label	N	Sex (M/F)	MeanAge	MeanFD
**ASD**	190	158/32	11.44	0.14
**TDC**	211	125/86	10.78	0.13
Movie TP				
Label	N	Sex (M/F)	MeanAge	MeanFD
**ASD**	214	183/31	11.21	0.1356
**TDC**	239	145/94	10.71	0.1252
Resting-State				
Label	N	Sex (M/F)	MeanAge	MeanFD
**ASD**	221	186/35	11.72	0.13
**TDC**	218	133/85	11.59	0.12

**Note:** Group differences in age and mean FD were assessed using two-sample t-tests, and differences in sex distribution were assessed using chi-square tests.
